# Spatial and Simultaneous Seroprevalence of Anti-*Leptospira* Antibodies in Owners and Their Domiciled Dogs in a Major City of Southern Brazil

**DOI:** 10.3389/fvets.2020.580400

**Published:** 2021-01-08

**Authors:** Aline do Nascimento Benitez, Thais Cabral Monica, Ana Carolina Miura, Micheline Sahyun Romanelli, Lucienne Garcia Pretto Giordano, Roberta Lemos Freire, Regina Mitsuka-Breganó, Camila Marinelli Martins, Alexander Welker Biondo, Isabela Machado Serrano, Thiago Henrique Carneiro Rios Lopes, Renato Barbosa Reis, Jancarlo Ferreira Gomes, Federico Costa, Elsio Wunder, Albert Icksang Ko, Italmar Teodorico Navarro

**Affiliations:** ^1^School of Medical Sciences and Institute of Computing, University of Campinas, Campinas, Brazil; ^2^Laboratory of Zoonoses and Public Health, Londrina State University, Londrina, Brazil; ^3^Centro Universitário Filadélfia - UniFil, Filadélfia University Center, Londrina, Brazil; ^4^Department of Preventive Veterinary Medicine, Londrina State University, Londrina, Brazil; ^5^Department of Nursing and Public Health, Ponta Grossa State University, Ponta Grossa, Brazil; ^6^AAC&T, Consultoria em Pesquisa Ltda, Curitiba, Brazil; ^7^Department of Veterinary Medicine, Federal University of Parana, Curitiba, Brazil; ^8^Department of Comparative Pathobiology, Purdue University, West Lafayette, IN, United States; ^9^Universidade Salvador, University of Salvador, Salvador, Brazil; ^10^Fiocruz, Gonçalo Moniz Research Institute, Brazilian Ministry of Health, Salvador, Brazil; ^11^Institute of Collective Health, Federal University of Bahia, Salvador, Brazil; ^12^Department of Epidemiology of Microbial Diseases, Yale School of Public Health, New Haven, CT, United States

**Keywords:** zoonosis, One Health, serovar, epidemiology, kernell analysis

## Abstract

Although leptospirosis has been considered a major concern in urban areas, no study to date has spatially and simultaneously compared both owner and dog serology in households of major cities. Accordingly, the aim of the present study was to assess the seroprevalence of *Leptospira* antibodies, evaluate associated risk factors and conduct spatial analyses in 565 randomly selected households, which included 597 dog owners and 729 dogs in Londrina, Southern Brazil. Seropositivity by MAT were detected in in 11/597 (1.84%) owners and in 155/729 (21.26%) dogs. The risk factors were evaluated with logistic regression analysis and spatial factors and case distribution were evaluated with kernel density analyses. The sera of 14/155 (9.03%) dogs reacted for more than one serovar with the same titer. Canicola was the most frequent serogroup, detected in 3/11 (27.27%) owners and 76/155 (49.03%) dogs. The highest titer among the owners was 1:3,200 and was detected in the same household with a titer of 1:800 in the dog. Simultaneous owner-dog seropositivity was found in 7/565 (1.23%) households, with three reacted against serogroup Canicola. Positive owners were detected in 4/565 (0.70%) households and positive dogs were detected in 141/565 (24.95%) households. The associated risks of infection for dogs were different from those associated with infection in owners. Risk analyses for Canicola also identified specific factors of infection. Regardless of owner and dog cases were not statistically clustered, the kernel map has shown dog positivity occurrence in the same hot locations and near positive owners. The dependent variable analysis and logit model suggested a greater likelihood of peri-domiciliary contact with *Leptospira*. In conclusion, exposure to *Leptospira* infection was significantly higher in dogs than in their owners and human cases spatially overlapped dog cases, implicating dogs as potential environmental sentinels for this disease. In addition, the associated risk may vary according to serogroup, and the observed simultaneous Canicola seropositivity of owner and dog has suggested intradomicile-transmitted infection.

## Introduction

Leptospirosis has been considered a worldwide emerging infectious and zoonotic disease caused by the spirochete *Leptospira* spp., which may persist for months in moist soil and water associated with the presence of *reservoir* animals in nature and accidentally transmitted to human beings (1). *Leptospiras* have been classified into over 300 pathogenic serovars (sv) according to structural antigenic characteristics and in 22 distinct genomospecies based on DNA-DNA hybridization composed of 10 pathogenic species, five intermediate and seven saprophytic species, but without correlations among those classifications. The genomic analysis is more accurate than serology during active infections, however, the serogroup identification by detection of anti-*Leptospiras* antibodies allows the identification of the animal *reservoir* (1, 2). Although the serovars reportedly adapted to specific animal species, such as sv Canicola for dogs, sv Bratislava for swine and sv Copenhageni for rats (3, 4) the association of serovars and mammal hosts has not been absolute, and their cellular and molecular basis remains to be fully established (5, 6).

In a leptospirosis surveillance study conducted from 1996 to 2005 in American countries, of which Brazil, Costa Rica, and Cuba have accounted for 83.1% of the 4,713.5 cases annually notified, Brazil alone has notified 3,165/4,717 (67.1%) cases and 349/380 (91.8%) deaths (7). Another systematic review with studies on leptospirosis incidence from 34 countries estimate that 1.03 million human cases and 58,900 deaths due to the disease have been reported annually, mostly concentrated in slums and other poor urban areas of developing countries (8). Disease endemicity and increased incidence have been mainly located in the Caribbean and Latin America, as well as in Southeast Asia and Oceania (9), despite leptospirosis has been considered endemic (restricted or peculiar to a locality or region) in other areas as well where flooding and other environmental conditions associated with rodent infestation may favor the *Leptospira* life cycle (10).

This pattern of human leptospirosis infection has mostly motivated studies either toward retrospectively confirmed cases (11–14) or socially vulnerable communities (7, 15–17). Although providing crucial information on leptospirosis infection and clinical onset, such contributions may not be epidemiologically extrapolated to other endemic regions located in the more prosperous urban areas of some developing countries (18). Not surprisingly, human leptospirosis cases still occur in areas with a high human development index (HDI) such as Londrina city (HDI: 0.841), northern Parana State (HDI: 0.790), Southern Brazil; this non-flooding urban area also has approximately one-fifth (132/653, 20.21%) seropositivity among the local dogs (19).

Still synanthropic rodents have been indicated as the main *Leptospira* reservoirs for human disease in urban settings (20, 21) the role played by dogs as sentinels or reservoirs has been controversial (22, 23). In this context, the World Health Organization (WHO) has demanded an increase in leptospirosis surveillance to determine global losses, improve surveillance methods and establish effective disease control and prevention (24). In addition, the WHO has called for studies focused on the One Health Initiative, combining human, animal and environmental health (25) in a holistic approach to zoonotic diseases (26).

To date, no study has spatially and simultaneously assessed and compared both owner and dog serology along with their household and correspondent risk factors in urban areas of major cities. Although molecular investigations which determine the evolutionary relationships of *Leptospira* infection between humans and dogs identifying and characterizing the circulating or infecting strains, serology has been a more sensitive indicator of past or present infection (3). Additionally, concomitant serology and spatial analyses performed with titration of human and dog samples may provide a better approach to the evaluation of risk factors, cross infection, and common household environmental exposure.

Accordingly, the aim of the present study was to assess the leptospirosis seroprevalence, the associated risk factors and conduct a spatial analysis in owners, dogs, and their respective households randomly selected of Londrina, a seat city of half-million people in Southern Brazil, which is nationally ranked 38th in population and 145th in human development index (HDI) out a total of 5,570 Brazilian cities.

## Materials and Methods

### Study Area and Population

The target population of this study was the residents from the urban area of Londrina (23°18′36″S and 51°09′46″W), the county seat of a metropolitan area and the second largest city of Parana State, Southern Brazil. Londrina was selected due to its high urban area of 97.00%, high human development index (HDI) of 0.841 (ranked 145th) and high urban population of 543,003 inhabitants (ranked 18th out of a total of 5,570 Brazilian cities). The city is located 608 meters above sea level with a rain forest biome under a subtropical humid climate; average temperatures range from 15.6 to 27.5° Celsius, with yearly average precipitation of 1,630 mm and average relative humidity of 71.10% (27, 28).

### Sample Size and Sampling

No data on the seroprevalence of anti-*Leptospira* antibodies were available at the time of the survey, either for human or dog general populations throughout the urban area. Thus, calculations for the sample size were designed with an expected 50% prevalence, 5% accuracy, 95% confidence level, and an initial population of 161,144 households [https://cidades.ibge.gov.br/v4/brasil/pr/Londrina/pesquisa/23/47427?detalhes=true&localidade1=410690] for a final minimum sampling size of 384 individuals, with visits distributed only in urban households using freely available software (EpiInfo 3.5.2, CDC, Atlanta, GA, USA). Inclusion criteria of at least one person and one dog per household were applied. Thus, a final minimum of 461 households was finally calculated due to the 20.0% safety margin of potential participation refusal, dog aggressiveness, inadequate sampling, closed household and commercial or public properties as stores, drugstores, parks, playgrounds, and schools.

The sample was randomly drawn by commercial software (BioEstat 3.0, Belém, PA, Brazil) (29). The sample included conglomerates of four households per block with a calculated total of 115 (461/4) blocks, two blocks per city section of urban planning and a total of 58 (115/2) city sections covered. The researchers were coordinated and guided by professionals from the City Secretary of Health office, which had previously informed the local neighborhoods about the visits, volunteer questionnaires and blood samplings. The inclusion criteria for owners included voluntarily signed informed consent, age 18 years or older, voluntary blood sampling by accredited nurses, and at least one dog in the same household. Domiciled dogs owned by household owner, dogs 6 months or older were eligible for inclusion.

An epidemiological questionnaire was applied to verify and avoid previous vaccination against canine leptospirosis.

Dog blood samples were obtained by a veterinarian following voluntarily signed informed consent by the dog owner. Aggressive dogs were not included for blood sampling due to city regulations on animal and human safety.

### Epidemiological Investigation

This was a cross-sectional study, and the risk of infection was investigated with an epidemiological questionnaire, which has been formulated, tested, and applied in previous studies (19). The questionnaires included closed questions on variables associated with owner and dog exposure to leptospirosis and were organized into three blocks: A. socioeconomic-environmental variables, B. personal sanitary habits and behavior, and C. animal behavior and management. The State Minimum Wage was R$ 880.00, equivalent to U$ 264.26 with an exchange rate of 3.33 for US$ Dollar to R$ Real at the time of survey.

### Serology

All blood samples were drawn between July 2015 and July 2016; the dog owners and their corresponding dogs were both sampled, and the questionnaires were completed in the same household on the same day. Serum samples were separated and stored at −20°C until they were tested by microscopic agglutination test (MAT), as previously described (5), against the serogroups Australis (serovar Bratislava), Autumnalis (serovar Butembo), Ballum (serovar Castellonis), Canicola (serovar Canicola), Grippothyphosa (serovar Grippothyphosa), Icterohaemorrhagiae (serovars Icterohaemorrhagiae and Copenhageni), Pomona (serovar Pomona), Pyrogenes (serovar Pyrogenes), and Sejroe (serovar Hardjo). Among the 200 available serovars for the MAT tests, the strains have been apparently the same in certain geographic regions. In the present study were selected the most prevalent serovars for human and dog cases in the study region in the past 6 years (19, 30, 31) and its availability as a bacterin.

Dog vaccines commercially available in Londrina city included Imunovet®(Biovet, São Paulo, Brazil), Vanguard plus® (Zoetis, New Jersey, USA), Vencomax 12® (Dechra, Northwith, UK), and Nobivac® (MSD, New Jersey, USA).

Since the present study aimed to compare human and dog exposure to leptospirosis, the selected profile of *Leptospira* live bacteria cultures for MAT was the same for both owner and dog samples. Sera were initially tested at a 1:100 dilution, and then those samples presenting positive agglutination were 2-fold diluted until their final titer (5). Thus, the predominant serogroup was defined as the serogroup with the maximum titer against its correspondent serovar.

Samples with the same titer for two or more serovars and samples from dogs vaccinated within 6 months of the sampling day were considered undetermined and excluded from the risk analyses (32, 33).

### Statistical Analysis

A descriptive analysis was conducted using the epidemiologic questionnaire variables based on general serogroup detection. A risk measure was used to assess the intensity of the association with risk factors (OR, odds ratio), and a chi-square test was performed to evaluate statistical significance. For the multivariate analysis, logistic regression models were performed with general serogroup detection as the dependent variable and the risk factors as the independent variables. The stepwise method was used to select the final models. To initiate the model processing, a cut-off *p* < 0.20 in the bivariate analysis was used, and the choice of better multivariate models was based on *p*-value (*p* < 0.05) and *r*-square (adjustments) for each independent variable, and the interpretation of final models was based on the adjusted ORs. A household was considered positive when at least one dog or one person is positive. The household positivity was analyzed to access the environmental intra domiciliary risk of infection for both owners and dogs.

Despite the 1-year duration of this study, the single household sampling methodology may have impaired the seasonality assessment. The ages of owners and dogs were tested for adherence to the normal distribution with the Shapiro-Wilk normality test. Both were asymmetric and not normally distributed, so to evaluate the difference between positive and negative samples, the Mann-Whitney *U*-test was used. These analyses were conducted in the “stats” package of the R environmental software program (34).

### Spatial Analysis

Points of data collection were determined by the current addresses, and maps with owner and dog case distributions were produced. In these maps, census sector data from IBGE database (free spatial database from Brazil) were also used [https://censo2010.ibge.gov.br/sinopseporsetores/], and flooding, green and water area data were obtained from official database from the city. The density of dog cases was evaluated with kernel density analysis to determine hotspots and compare with potential clusters of owner cases. Flooding and water areas were also concomitantly plotted on the maps to evaluate their spatial association with the data. Despite the effect of green and water areas have not assessed through the regression analysis, flooding has been included as accumulated water in the regression analysis These spatial analyses were conducted using R software environmental with the “epiDisplay,” “spatstat,” and “maptools” packages (35, 36).

The first step was to estimate a logit without considering any spatial effects. Residues of logistic regression have shown spatial correlation. Moran's I test applied for testing whether residuals of regressions were spatially clustered, with a statistically significant value of 0.09 (*p* = 0.002) for a matrix of weight with the nearest neighbor. Such outcome requested a spatial analysis. Several spatial weight matrices were tested to verify whether the regression residues had significant Moran's I statistics.

Following, a multivariate analysis of spatial regression has been applied to identify variables explaining prevalence of leptospirosis in dogs. Independent variables included a dummy to register the presence of any reagent human to leptospirosis in the household (Presence of a positive human); whether the dog was vaccinated within the last 6 months (Vaccine); whether the dog had outdoors access (Street Access) or with other dogs inside the household (More than one dog in the house); number of dogs living in the household (Presence of dogs); dummies capturing income range of dog owners (Income2 and Income3); number of people living in the household (Households). Important to mention that the spatial multivariate model had a different specification from the first logistic models, with some very highly correlated variables.

In addition to the above independent variables, two factors were also added (FACT_1 and FACT_2) which represented a linear combination of variables with strong multi-colinearity, including (i) dummy indicating presence of wasteland near the household (Wasteland); (ii) dummy indicating whether the household has outside bathroom (Bathroom outside); (iii) number of rats seen at the yard (Rats); (iv) frequency of yard cleaning (Clean backyard); (v) dummy indicating whether yard had rats (Rats_at backyard); (vi) trash seen at the yard (Dirty backyard); and (vii) whether yard had rubble (Trash at backyard). Factorial analysis was applied to test the above factors.

To calculate the factors from factor analysis, was used to calculate the tetrameric correlations by the maximum likelihood estimator (iterative) obtained from the bivariate probit, using the Edwards and Edwards estimator as the initial value (37). The uniqueness was tested to verify how much of the common variance each variable may represent. In other words, high uniqueness may suggest that the extracted factors may have described the variables well. The results of the factorial analysis made it possible to transform the seven variables mentioned above into two factors (FACT_1 and FACT_2) according to Table 1.

**Table 1:** Matrix of components and commonality of indicators.

**Variable**	**Factor 1**	**Factor 2**	**Commonality**
Wasteland	0.16	-	0.97
Bathroom outside	-	0.29	0.90
Rats	-	0.72	0.27
Clean backyard	0.66	-	0.36
Rats_at backyard	-	0.54	0.70
Dirty backyard	0.93	-	0.04
Trash at backyard	0.87	-	0.08

As the errors of logistic regression showed a special correlation, it was important to estimate an econometric model taking into account the space so as not to omit a relevant variable.To incorporate a term of the spatially lagged dependent variable into the explanatory variables, the spatial autoregressive model (SAR) estimated by means of maximum likelihood and generalized method of moments was used. The SAR model can be specified as:

(1) $$\begin{array}{l} y_{t}=\rho Wy_{t}+X_{t}\beta +\varepsilon_{t} \end{array}$$

where ρ is the auto-regressive lag parameter (−1 < ρ <1) and $Wy_{t}={\left(Wy_{1t},\ldots ,Wy_{Nt}\right)}^{\prime }$ is the vector of the lagged dependent variable; $X_{t}={\left({X_{kt}}^{\prime },\ldots ,{X_{Nt}}^{\prime }\right)}^{\prime }$ is a matrix of observations of explanatory variables and $\beta ={\left(\beta_{1},\ldots ,\beta_{k}\right)}^{\prime }$ it is a vector of parameters to be estimated.

A second group of models was called the spatial error model (SEM), where the spatial dependence was considered residual and represented by the first-order autoregressive structure in the error term (37). The SEM model can be expressed as follows:

(2) $$\begin{array}{l} y_{t}=X_{t}\beta +\xi_{t} \end{array}$$

(3) $$\begin{array}{l} \xi_{t}=\lambda W_{2}\xi_{t}+\varepsilon_{t} \end{array}$$

In which ε is a multivariate normal distribution with zero mean and covariance matrix σ^2^*I*; the coefficient λ represents the parameter of the spatial autoregressive error. In the SEM model, errors represent an average of errors in neighboring regions plus a component of random error.

The presence of the spatially-lagged dependent variable (Wy) was equivalent to the introduction of an endogenous variable, using the ordinary least squares method as previously described (38). All estimates were presented to identify the variable robustness.

**Figure 1:**
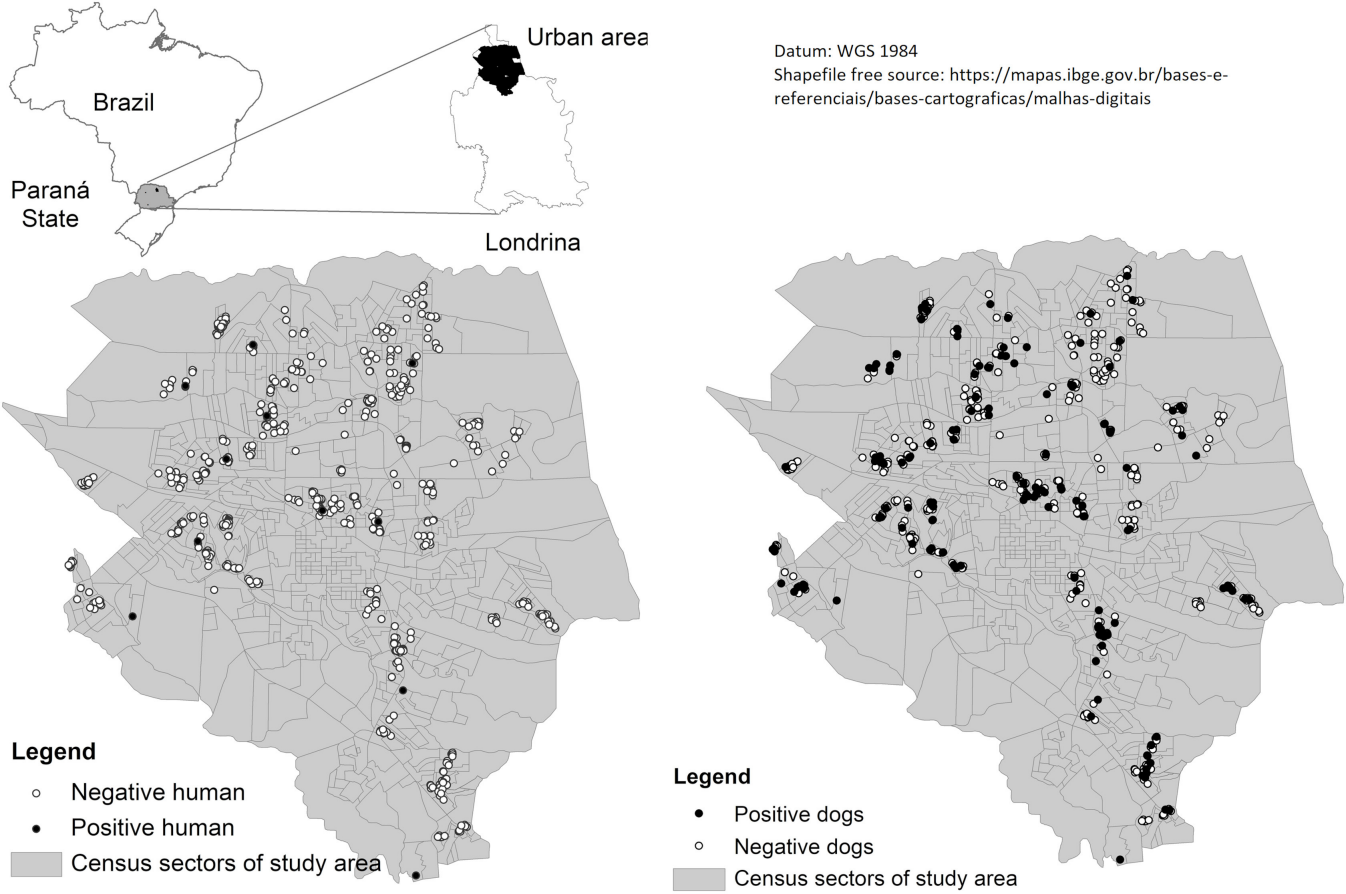
Distribution of owners and dogs positive and negative for *Leptospira* from July 2015 to July 2016 in the urban area of Londrina, Southern Brazil.

**Figure 2:**
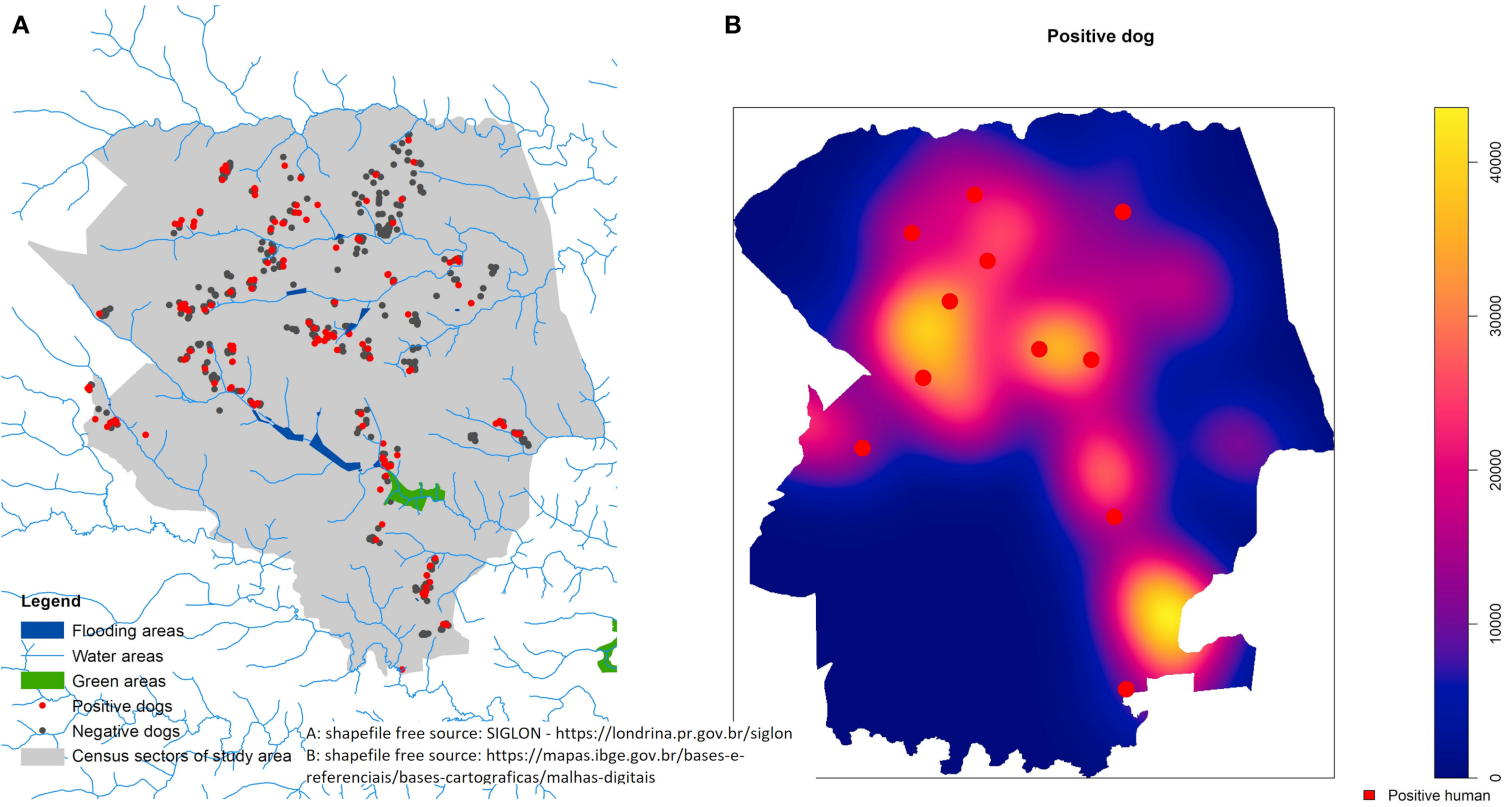
Distribution of positive dogs in flooding, green and water areas **(A)** and kernel density analysis for positive dogs with overlapping positive owner locations **(B)** from July 2015 to July 2016 in the urban area of Londrina, Southern Brazil.

A map illustrating the municipality of sampling of the studied regions (source: free access Brazilian databases https://downloads.ibge.gov.br/downloads_geociencias.htm) was produced by authors, using these free open access shapefiles and performed on GIS software using ArcGIS 10 and presented (Figures 1, 2).

### Ethical Aspects

This study was approved by the National Human Ethics Research Committee (protocol number 1,025,861/2014) and the Animal Use Ethics Committee (protocol number 181/2014), both at Londrina State University, Southern Brazil. In addition, the present study was approved by the Londrina City Secretary of Health and was officially included as part of the annual activities. In addition, all interventions were authorized by the Human Beings Ethics Studies Committee (protocol number 1,025,861) and the Animal Use Ethics Committee of the State University of Londrina (protocol number 181/2014).

## Results

A total of 750 households were visited, and the minimum sample size calculation was surpassed with 565/461 (122.56%) households; a total of 597/1,985 (30.07%) owners and 729/1,170 (62.30%) dogs sampled. Overall, 11/597 (1.8%) owners and 155/729 (21.3%) dogs were identified with anti-*Leptospira* titers by MAT, which represented 141/565 (25.0%) of the sampled households (Table 2).

**Table 2:** Distribution of 597 houses with owners and/or dogs seropositive for *Leptospira*, Canicola serovar, or other serogroups, from July 2015 to July 2016 in the urban area of Londrina, Southern Brazil.

**Prevalences**	**Owners**		**Dogs**		**Houses**	
	***n*/total (%)**	**95% CI**	***n*/total (%)**	**95% CI**	***n*/total (%)**	**95% CI**
*Leptospira*	11/597 (1.8)	0.8–3.0	155/729 (21.3)	18.4–24.4	141/565 (25.0)	21.5–28.3
Canicola serogroup	3/597 (0.5)	0.0–1.2	76/729 (10.4)	8.2–12.9	70/565 (12.4)	9.9–15.0
Other serogroups	8/597 (1.3)	0.5–2.3	79/729 (10.8)	8.5–13.2	71/565 (12.6)	9.9–15.8

Canicola was the most frequently reactive serogroup in dogs, with titers identified in 76/155 (53.9%) samples, followed by serogroups Autumnalis and others with 65/155 (41.9%) sample positivity. On the other hand, Autumnalis was the most frequent serogroup in owners, found in 4/11 (36.36%) positive samples, followed by Canicola in 3/11 (27.27%) positive samples and other serogroups found in 4/11 (36.36%) positive samples (Table 3).

**Table 3:** Antibody titers for pathogenic *Leptospira* serogroups in positive serum samples of 11 owners and 141 dogs from July 2015 to July 2016 in the urban area of Londrina, Southern Brazil.

		**Antibodies titers**								
**Serogroup**	**Serovar**	**100**	**200**	**400**	**800**	**1,600**	**3,200**	**6,400**	**12,800**	**Total (%)**
**Owner samples**										
Autumnalis	Butembo	04	-	-	-	-	-	-	-	4 (40.0)
Canicola	Canicola	-	01	-	02	-	-	-	-	3 (30.0)
Grippothyhosa	Grippothyphosa	01	01	-	-	-	-	-	-	2 (20.0)
Ballum	Castellonis	01	-	-	-	-	-	-	-	1 (10.0)
Icterohaemorrhagiae	Icterohaemorrhagiae	01	-	-	-	-	-	-	-	1 (10.0)
**Total**		07	02	-	02	-	-	-	-	10 (100.0)
**Dog samples**										
Canicola	Canicola	21	21	09	09	10	04	01	01	76 (53.9)
Autumnalis	Butembo	08	05	07	-	01	-	-	-	21 (14.9)
Australis	Bratislava	09	03	-	-	-	-	-	-	12 (8.5)
Grippothyhosa	Grippothyphosa	05	02	01	01	-	-	-	-	09 (6.4)
Icterohaemorrhagiae	Copenhageni	03	03	01	-	-	-	-	-	07 (5.0)
Icterohaemorrhagiae	Icterohaemorrhagiae	03	-	-	-	-	-	-	-	03 (2.1)
Pomona	Pomona	01	05	01	-	-	-	-	-	07 (5.0)
Pyrogenes	Pyrogenes	02	02	-	-	-	01	-	01	06 (4.3)
**Total**		52	41	19	10	11	05	01	02	141 (100.0)

In 70/141 (49.64%) households, either owners or dogs were reactive to serogroup Canicola, and 71/141 (50.35%) were reactive to at least one of the tested serogroups (Table 2). The highest titers were 1:12,800 for dogs and 1:800 for owners, both to serogroup Canicola; other serogroups reached an equally high titer of 1:1,600 for dogs, but the highest titer was 1:200 for dog owners (Table 3).

Simultaneous dog owner and dog seropositivity was found in 7/565 (1.23%) households, of which three were reactive for serogroup Canicola in owners and dogs, and different serogroups were observed in four households. There were 4/565 (0.70%) households that had only owner-positive samples, and only one dog-only positive household was detected among the total 141/565 (24.95%) positive households (Table 4).

**Table 4:** Antibody titers against pathogenic *Leptospira* serogroups in the samples from the 11 households with positive dog owners from July 2015 to July 2016 in the urban area of Londrina, Southern Brazil.

**House**	**Owners**		**Dogs**		**House**	**Owners**		**Dogs**	
	**Serogroup**	**Titer**	**Serogroup**	**Titer**		**Titer**	**Titer**	**Serogroup**	**Titer**
A	Canicola	800	Canicola	3,200	G	Autumnalis	100	Canicola	100
B	Canicola	800	Canicola	1,600	H	Autumnalis	100	Negative	-
C	Canicola	200	Canicola	3,200	I	Autumnalis	100	Negative	-
D	Grippotyphosa	100	Autumnalis	200	J	Ballum	100	Negative	-
E	Grippotyphosa	200	Autumnalis	100	K	Icterohaemorrhagiae	100	Negative	-
F	Autumnalis	100	Grippotyphosa	100					

For owners, the bivariate analysis of risk factors associated with *Leptospira* antibodies was statistically significant for houses with positive dogs (*p* = 0.021) and houses with nearby forest (*p* = 0.043). The multivariate logistic regression with owners positive for *Leptospira* as the dependent variable did not produce a significant model (Table 5).

**Table 5:** Aspatial logistic regression applied to variables with owners seropositivity to leptospirosis.

	**Variables**		**Positive *n* (%)**	**Negative *n* (%)**	**Total *N***	**OR**	**95% CI**	***p*-value**
	**Owner**							
^*^	Gender	Female	5 (1.1)	434 (98.9)	439	0.35	0.10-1.23	0.095
		Male^R^	5 (3.2)	153 (96.8)	158			
^*^	Income	<1 MW	0 (0.0)	147 (100.0)	147	1.02	1.01-1.04	0.058
		> 1 MW^R^	10 (2.2)	440 (97.8)	450			
	Accumulated water	Yes	1 (1.3)	78 (98.7)	79	0.73	0.09-5.80	0.610
		No^R^	9 (1.7)	509 (98.3)	518			
	Open sewage	Yes	1 (2.5)	39 (97.5)	40	1.56	0.19-12.6	0.503
		No^R^	9 (1.6)	548 (98.4)	557			
	Exposed garbage	Yes	7 (1.6)	419 (98.4)	426	0.94	0.24-3.66	0.582
		No^R^	3 (1.8)	168 (98.2)	171			
	Wasteland	Yes	6 (2.0)	299 (98.0)	305	1.44	0.40-5.17	0.403
		No^R^	4 (1.4)	288 (98.6)	292			
^*^	Bathroom outside	Yes	4 (3.5)	111 (96.5)	115	2.85	0.79-10.26	0.108
		No^R^	6 (1.2)	474 (98.8)	480			
^*^	Presence of rats	Yes	5 (1.1)	446 (98.9)	451	0.32	0.09-1.11	0.070
		No^R^	5 (3.4)	141 (96.6)	146			
^**^	House with postive dog	Yes	6 (3.9)	146 (96.1)	152	4.52	1.26-16.24	0.021
		No^R^	4 (0.9)	440 (99.1)	444			
	Dirty backyard	Yes	4 (1.7)	234 (98.3)	238	1.01	0.28-3.60	0.616
		No^R^	6 (1.7)	353 (98.3)	359			
^*^	Job outside	Yes	1 (0.5)	210 (99.5)	211	0.20	0.03-1.58	0.080
		No^R^	9 (2.3)	375 (97.7)	384			
^**^	Nearby forest	Yes	1 (0.4)	240 (99.6)	241	0.16	0.02-1.28	0.043
		No^R^	9 (2.5)	347 (97.5)	356			
	Icterus as clinical sign	Yes	1 (2.0)	48 (98.0)	49	1.33	0.16-10.82	0.560
		No^R^	9 (1.5)	509 (98.5)	517			

Bivariate and multivariate logistic regression analysis of dog owners positive for Leptospira from July 2015 to July 2016 in the urban area of Londrina, Southern Brazil.

* Variables included in the logistic models.

R Reference category.

** Variables with p < 0.05.

There was no significant multiple logistic model.

MW, minimum wage.

For dogs, the analysis of risk factors associated with *Leptospira* antibodies was statistically significant for exposed garbage (*p* = 0.030), male sex (*p* = 0.003), presence of equines (*p* = 0.001), presence of opossums (*p* = 0.032), and nearby forests (*p* = 0.017) (Table 6). The multivariate logistic regression with dogs positive for *Leptospira* as the dependent variable produced a significant model, with the presence of equines (*p* < 0.001, OR 0.19), female sex (*p* = 0.019, OR 1.67), and exposed garbage (*p* = 0.041, OR 1.51) (Table 6).

**Table 6:** Aspatial logistic regression applied to variables with dogs seropositivity to leptospirosis.

	**Variables**		**Positive *n* (%)**	**Negative *n* (%)**	**Total *N***	**OR**	**95% CI**	***p*- value**
	**Dogs**							
	Income	≤ 1 MW	37 (21.9)	132 (78.1)	169	1.05	0.69–1.59	0.447
		> 1 MW^R^	118 (21.1)	442 (78.9)	560			
^*^	Accumulated water	Yes	25 (26.0)	71 (74.0)	96	1.36	0.83–2.24	0.137
		No^R^	130 (20.5)	503 (79.5)	633			
	Open sewage	Yes	12 (21.1)	45 (78.9)	57	0.99	0.51–1.91	0.562
		No^R^	143 (21.3)	529 (78.7)	672			
^**^	Exposed garbage	Yes	104 (19.4)	431 (80.6)	535	0.68	0.46–0.99	0.030
		No^R^	51 (26.3)	143 (73.7)	194			
	Wasteland	Yes	80 (21.3)	296 (78.7)	376	1.00	0.70–1.43	0.532
		No^R^	75 (21.2)	278 (78.8)	353			
^**^	Sex	Female	71 (17.4)	336 (82.6)	407	1.49	1.13–1.97	0.003
		Male^R^	84 (26.1)	238 (73.9)	322			
	Bathroom outside	Yes	28 (20.7)	107 (79.3)	135	0.97	0.61–1.53	0.493
		No^R^	126 (21.3)	465 (78.7)	591			
	Presence of rats	Yes	116 (20.6)	446 (79.4)	562	0.85	0.56–1.29	0.258
		No^R^	39 (23.4)	128 (76.6)	167			
^*^	Street Access	Yes	93 (24.0)	294 (76.0)	387	1.43	0.99–2.05	0.052
		No^R^	62 (18.1)	280 (81.9)	342			
	Hunting Habit	Yes	69 (21.6)	250 (78.4)	319	1.04	0.73–1.49	0.830
		No^R^	86 (21.0)	324 (79.0)	410			
^**^	Presence of equines	Yes	15 (60.0)	10 (40.0)	25	6.04	2.66–13.74	0.001
		No^R^	140 (19.9)	564 (80.1)	704			
^*^	Presence of bovines	Yes	2 (66.7)	1 (33.3)	3	7.49	0.67–83.15	0.116
		No^R^	153 (21.1)	573 (78.9)	726			
^**^	Presence of opossums	Yes	3 (75.0)	1 (25.0)	4	11.31	1.17–109.49	0.032
		No^R^	152 (21.0)	573 (79.0)	725			
^***^	Presence of other positive dogs	Yes	0 (0.0)	50 (100.0)	50	-	-	-
		No^R^	155 (22.8)	524 (77.7)	679			
^*^	Clinical sign: vomit and/or diarrhea	Yes	21 (17.1)	102 (82.9)	123	0.73	0.44–1.20	0.129
		No^R^	134 (22.1)	472 (77.9)	606			
	Dirty backyard	Yes	62 (20.3)	244 (79.7)	306	0.90	0.63–1.29	0.320
		No^R^	93 (22.0)	330 (78.0)	423			
^**^	Nearby forest	Yes	33 (29.5)	79 (70.5)	112	1.69	1.08–2.66	0.017
		No^R^	122 (19.8)	495 (80.2)	617			
	Contact with other domestic animal	Yes	124 (21.0)	467 (79.0)	591	0.92	0.59–1.43	0.390
		No^R^	31 (22.5)	107 (77.5)	138			
	Presence of dogs	Yes	115 (20.9)	434 (79.1)	549	0.93	0.62–1.39	0.395
		No^R^	40 (22.2)	140 (77.8)	180			
	Clinical sign: weight loss	Yes	16 (23.9)	51 (76.1)	67	1.18	0.65–2.13	0.583
		No^R^	139 (21.0)	523 (79.0)	662			
**Final logistic model**		**Adjusted-OR**		**95 CI adjusted-OR**		***p*****-value (Wald test)**		
Presence of equines		0.19		0.08–0.43		<0.001		
Sex (female)		1.67		1.17–2.23		0.019		
Exposed garbage		1.51		1.02–2.23		0.041		

Bivariate and multivariate logistic regression analysis of dogs positive for Leptospira from July 2015 to July 2016 in the urban area of Londrina, Southern Brazil.

There was no significant interactions between co-variates of the final model.

* Variables included in the logistic models.

** There was no sufficient expose and no expose to proceed the analysis.

*** There was no sufficient animals to calculate.

MW, minimum wage.

R Reference category.

For households, the analysis of risk factors associated with *Leptospira* antibodies showed statistical significance for open sewage (*p* = 0.014). The multivariate logistic regression with households positive for *Leptospira* as the dependent variable did not produce a significant model (Table 7).

**Table 7:** Aspatial logistic regression applied to variables with households positivity to leptospirosis.

	**Variables**		**Positive *n* (%)**	**Negative *n* (%)**	**Total *N***	**OR**	**95% CI**	***p*-value**
	**House**							
	Income	≤ 1 MS	40 (27.8)	104 (72.2)	144	1.22	0.79–1.87	0.212
		> 1 MS^R^	101 (24.0)	320 (76.0)	421			
	Accumulated water	Yes	17 (22.1)	60 (77.9)	77	0.83	0.47–1.47	0.318
		No^R^	124 (25.4)	364 (74.6)	488			
^**^	Open sewage	Yes	4 (10.0)	36 (90.0)	40	0.32	0.11–0.90	0.014
		No^R^	137 (26.1)	388 (73.9)	525			
	Exposed garbage	Yes	103 (25.1)	308 (74.9)	411	1.02	0.67–1.57	0.509
		No^R^	38 (24.7)	116 (75.3)	154			
^*^	Wasteland	Yes	80 (26.7)	220 (73.3)	300	1.22	0.83–1.79	0.183
		No^R^	61 (23.0)	204 (77.0)	265			
	Bathroom outside	Yes	25 (22.9)	84 (77.1)	109	0.87	0.53–1.42	0.333
		No^R^	116 (25.6)	338 (74.4)	454			
^*^	Presence of rats	Yes	98 (23.1)	327 (76.9)	425	0.68	0.44–1.03	0.069
		No^R^	43 (30.7)	97 (69.3)	140			
	Dirty backyard	Yes	58 (25.1)	173 (74.9)	231	1.01	0.69–1.49	0.944
		No^R^	83 (24.9)	251 (75.1)	334			

Bivariate and multivariate logistic regression analysis of households positive for Leptospira from July 2015 to July 2016 in the urban area of Londrina, Southern Brazil.

* Variables included in the logistic models.

** Variables with p < 0.05.

MW, minimum wage.

There was no significant logistic model.

R Reference category.

As can be seen in Table 8, the results demonstrate that intrahousehold conditions, including the backyard situation, rats and family income, have not presented significant effects for dog infection and have failed to explain the probability of a dog infected by *Leptospira* in the household, while parameters related to the neighborhood were significant for dog infection. Dogs from households with unprotected bag discharge in the current study were more likely (and confirmed by logistic model) to be infected by *Leptospira* and serogroup Canicola, while parameters related to the neighborhood were significant for dog infection.

**Table 8:** Spatial multivariate logistic regression.

	**Multivariate logistic**	**MV (SAR)^**b**^**	**MV (SEM)^**c**^**	**GMM (SAR)^**d**^**
**Dependent variable: dogs positive to** ***Leptospira***				
Presence of a positive human	1.48^***^	0.24^**^	0.26^***^	0.23^**^
	(0.50)	(0.10)	(0.10)	(0.12)
Vaccine	0.01	−0.09^*^	−0.09^**^	−0.09^**^
	(0.07)	(0.05)	(0.05)	(0.04)
Street Access	0.09	0.10^**^	0.11^***^	0.10^**^
	(0.10)	(0.04)	(0.04)	(0.04)
Income2	−0.12	−0.01	0.00	−0.01
	(0.23)	(0.04)	(0.04)	(0.04)
Income3	−0.11	0.03	0.04	0.03
	(0.27)	(0.04)	(0.05)	(0.04)
Presence of dogs	−0.06	−0.06	−0.05	−0.06
	(0.06)	(0.04)	(0.04)	(0.04)
More than one dog in the house	−0.06	0.00	0.01	0.00
	(0.26)	(0.01)	(0.01)	(0.01)
FACT_1	−0.01	−0.08	−0.07	−0.07
	(0.22)	(0.05)	(0.05)	(0.05)
FACT_2	−0.01	−0.07	−0.06	−0.06
	(0.33)	(0.05)	(0.05)	(0.05)
Households	0.02	0.01	0.01	0.01
	(0.05)	(0.01)	(0.01)	(0.01)
Constant	−1.29^***^	0.19^***^	0.22^***^	0.15^***^
	(0.32)	(0.06)	(0.06)	(0.07)
Lambda		0.02^***^		0.05^***^
		(0.01)		(0.02)
Rho			0.03^***^	
			(0.01)	
AIC^a^	761	757	751	-
BIC	811	822	816	-

a Akaike's information.

b Maximum Likelihood Estimation.

c Maximum Likelihood Estimation.

d Estimation by the Generalized Method of Moments because of the endogeneity of the spatially lagged dependent variable.

*** Variables with p < 0.01.

** Variables with p < 0.05.

* Variables with p < 0.10.

The spatial analysis is shown in Figures 1, 2 and demonstrated a visual overlap between dog and owner positive cases (Figure 2B).

**Figure 3:**
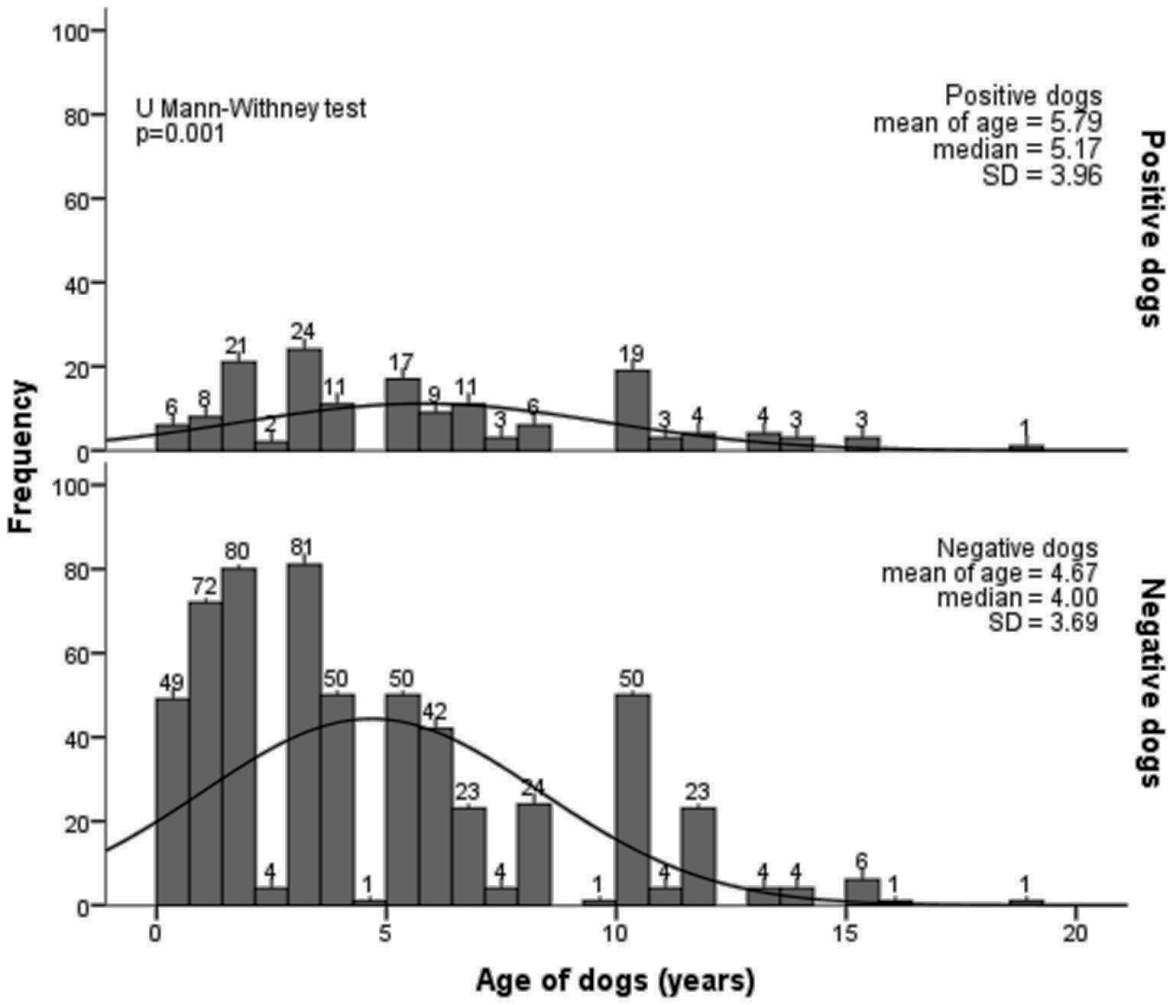
Histogram of age for positive and negative dogs for *Leptospira* from July 2015 to July 2016 in the urban area of Londrina, Southern Brazil.

The age analysis showed no significant differences between positive (53.44 ± 18.15 years) and negative (50.87 ± 17.16 years) dog owners (*p* = 0.60). For dogs, the age of positive dogs (5.79 ± 3.96) was significantly higher than that of negative dogs (4.67 ± 3.69) (*p* = 0.001). This variable was included in multilevel regression analysis, but lost significance when with others (Figure 3).

The final logistic and spatial regressions were obtained after testing whether the factorial analysis could be applied to transform highly correlated variables in few factors (Supplementary File). It was possible to build two factors that together explained 60.2% of the total data variance. The loss of information was relatively low, and synthetic indicators based on factor analysis may have contained the appropriate characteristics. The spatial model, which has used a dependent variable with a variable dummy indicating a positive leptospirosis dog, included two factors (FACT_1 e FACT_2). The factor 1 explaining 38.5% of variance was more correlated to yard variables such as trash, rubble, and low cleaning frequency. The factor 2 explaining 21.7% was more associated to presence and observation of rats. These two factors were also included in the spatial regression analysis besides the control variables described on section “spatial analysis.”

After testing 15 matrices of different weights, the results have shown that the residuals were more strongly correlated with the contiguity matrix of the nearest neighbor. Thus, the spatial model has considered this weight matrix and outcome of spatial models were analyzed and presented (Table 8).

## Discussion

The serological approach to the evaluation of simultaneous and spatial *Leptospira* antibodies in owners and their dogs was accomplished for the first time by the present study, with an overall human:dog leptospirosis positivity ratio of 1:11.55 and an owner seroprevalence that was significantly lower than that of their dogs. Using a similar comprehensive approach, our research group previously demonstrated the opposite pattern for toxoplasmosis, with a human:dog ratio of 2.55:1, an owner seroprevalence significantly higher than that of their dogs, with canine seroprevalence directly associated with having more dogs and a dirty backyard, and with spatial differences between owner and dog exposures (39).

Serological surveys on canine leptospirosis throughout Latin America have shown wide-ranging prevalence rates, varying from 4.9 to 72.0% depending on country, region, dog population and historical endemic level (40). Prevalence studies have varied from 41/335 (12.23%) positive stray dogs in northern Brazil (40), 163/1,233 (13.21%) positive domiciled dogs in a poor flooding area in eastern Brazil (23), 35/175 (20.00%) positive culled stray dogs in western Brazil (18), 132/653 (20.21%) positive owned dogs in an urban area near Londrina (19), 7/33 (21.21%) positive abandoned stray dogs on the Londrina State University campus (30), 51/236 (21.61%) positive owned dogs from a University neutering program in northern Brazil (31), and 33/228 (14.4%) and 35/90 (38.9%) positive dogs in the same city of the capital metropolitan area in an eastern Brazilian (41) state.

The seroprevalence of 155/729 (21.26%) positive dogs in the current study was within previous findings for Londrina city (20.21, 21.21, and 21.61%), with surprisingly non-significant differences in prevalence despite differences in the dogs with regard to street access and owner care (18, 19, 30, 31). Thus, the current study may offer a comprehensive and non-biased serologic survey of domiciled dogs throughout the urban city area by randomly including dogs and owners from a representative household distribution.

The Brazilian Ministry of Health has established a unified mandatory notification system for suspected human leptospirosis cases, which provides epidemiological information on endemicity nationwide. Despite subpar notification rates due to lack of diagnosis and mild or non-attended cases, Parana was ranked fifth out of 26 Brazilian states and the national capital in 2015, with 362/3,257 (11.11%) of the total human confirmed cases, of which 18/362 (4.97%) cases and 07/18 (38.88%) deaths were reported in Londrina; a similar pattern with 05/14 (35.71%) deaths was observed in 2016 (42). The human seroprevalence results of 11/597 (1.84%) in the current study may corroborate the only two human studies from the same region, which have found 25/207 (12.1%) human cases near (33 km) Londrina city (33) and 2/157 (1.27%) cases among veterinary students in the northwestern Parana State (43). Despite the contact with positive dogs, the frequency of human infection and infection risk have been relatively low and the simultaneous positive serology of owners and dogs has provided a comparative and statistically significant human:dog ratio of 2.55:1, which may be used as a comparative parameter of local exposure to *Leptospira*.

Differences in human and dog serology may reflect distinct infection patterns according to host species. While pathogenic *Leptospira* have mostly caused human acute disease by accidental host infection without renal carrier status (1), dogs present different degrees of acute or chronic disease and occasional colonization of the renal tubules, leading to a long-term shedding and reservoir state (3). In such a scenario, a higher prevalence of seropositive dogs in a specific area may indicate spirochete circulation among animal populations, occasionally leading to human infection (44). Molecular investigations in different hosts have shown that the genetic machinery of serogroup Canicola may lead to a similar infection potential in human beings (45), pigs and dogs (46).

The present study has shown that seropositive domiciled dogs may indicate an intra- and peridomiciliar risk environment because they were exposed daily to the outdoor area near the household environment, returning at night, exponentially increasing contact and potentiate owner infection. Although eliminating outside may not directly characterize an associated risk factor for leptospirosis in dogs, the likelihood of rats in the backyard may increase under such conditions.

Although rodents have been considered the main urban hosts for leptospiral harboring and maintenance, particularly in slums (16, 47), dogs and other animal species may be implicated in the local epidemiology of human disease (48). Leptospiral genotyping in human and rat infections in Seychelles, which has one of the highest worldwide incidence rates, has proposed other animal reservoirs (49). In addition, a space-time association has been established between domestic animal and human incidence, with the epidemiology of animal infection being an associated risk for local human infection (50).

Although the present study has focused on concomitant seroprevalence and associated risk factors for leptospirosis seropositivity, individual analysis of serogroups, particularly Canicola, may provide important information since the role of dogs were surveyed as potential reservoirs and as susceptible species. Such a double role of dogs in the leptospiral life cycle may lead to long periods of infection and may explain the higher prevalence of serogroup Canicola in 3/11 (27.27%) owners and 76/155 (49.03%) dogs. However, detection of other serogroups in 8/11 (72.72%) owners and 65/155 (41.94%) dogs may indicate the presence of other environmental reservoirs that may be a source of infection for both human beings and dogs.

A previous survey of human and animal leptospirosis in Southern Brazil (51) found Canicola to be the most prevalent serogroup in dogs with 329/1,176 (27.96%) positive for the Tande strain, 266/1,176 (22.60%) positive for the Kito strain and 216/1176 (18.34%) positive for the Hond Utrecht IV strain; Autumnalis was the most prevalent human serogroup, with 195/997 (19.41%) seropositive humans. A previous study similarly found low detection of the common worldwide human serogroup Icterohaemorrhagiae (Copenhageni and Icterohaemorrhagiae) among human beings and dogs (46, 52).

Early studies have molecularly detected shedding of Leptospira in the urine of asymptomatic dogs with different serological titers (53, 54). In addition, MAT may not differentiate among infection, vaccination, and maternal antibodies (55), and puppies younger than 6 months and dogs vaccinated dogs <6 months prior were excluded from the descriptive statistical analyses. Hence, leptospiruria in any given dog may have played a role in environmental contamination in the present study. In addition, due to the lack of paired samples, particularly from seropositive titers, no disease could be confirmed based on a 4-fold increase in titer between paired sera (56). For dogs, parameters defining infection have not been fully established. Thus, although human titers ≥ 400 for one or more serogroups can be interpreted as a present or recent infection, no extrapolation has been made for dogs.

Despite it was not the most frequent, the high frequency of serogroup Autumnalis and the decreased frequency of serogroup Icterohaemorrhagiae have corroborated previous studies; this pattern may be associated with long-term canine vaccination and may have demonstrated a distinct pattern of leptospirosis, which may suggest urban environmental contamination (57). Although not the focus of the present study, rodents, and other local animal species (opossums, agoutis, capybaras) should be further surveyed, if possible, to fully establish their role regarding each leptospiral serogroup. Such studies should be used as a basis for future local public health actions for leptospirosis control and prevention.

Considering that human leptospirosis may cause non-specific febrile disease and self-remission within a week after onset (58), the three owners with titers for serogroup Canicola of 1:200, 1:200, and 1:800 may have experienced mild infection since no clinical signs were mentioned at the time of blood samplings. Since the dogs of these owners also presented high titers, with 1:3,200, 1:1,600, and 1:3,200 for serogroup Canicola, respectively, the same exposure source in the intra-domiciliary infection from dog to its owner should be considered. The current analysis detected a statistically significant association between the presence of a reactive dog in the household and a greater likelihood of infection by *Leptospira* in its owner. Further studies should focus on the serological and molecular assessment of dogs, dog owners, rats, and the environment in the same household to fully establish the role of each on the *Leptospira* life cycle.

The association between households with a seropositive owner or dog and risk of infection may also suggest the intra-domiciliary influence on infection for both owners and dogs. Moreover, proximity among households with positive owners from households with positive dogs has suggested the likelihood of peri-domiciliary infection. Unexpectedly, no clusters were observed in the studied area, and peri-domiciliary standing water following rain, green areas and water areas were not associated with the likelihood of infection; these factors have been previously shown to favor the survival of pathogenic *Leptospira* (59).

In the present study, despite the impossibility of multivariate logistic model calculation in owners due to the low prevalence of 11/597 (1.84%) positive individuals, the univariate analysis showed an association between visiting woody areas and *Leptospira* infection; however, there was no association between infection and having these areas near the residences. Hence, even non-endemic and no-flooding areas may be exposed to infection due to other environmental causes. In environments of high infection risk due to rodent infestation and flooding, a decrease in human leptospirosis cases may be reached by efforts in community improvements, particularly at the household and individual levels (60). Likewise, dogs from households with unprotected bag discharge were more likely (and confirmed by logistic model) to be infected by *Leptospira* and serogroup Canicola in the current study. Exposed garbage outside of the households may have attracted rats, peridomestic and wild species and also stray dogs nearby and contributed to the environmental contamination with the *Leptospira* in the surrounding microenvironments; a similar finding was previously observed in a case-control outbreak of human leptospirosis in which the presence of seroreactive dogs with leptospiruria in an owner-case household may have suggested high environmental contamination that caused a sequence of direct transmission (61).

The association of female dogs with anti-*Leptospira* antibodies has not been corroborated by previous studies, which have shown males with higher prevalence than females, probably due to territorial demarcation (62). However, the prior study was performed in stray dogs, and different degrees of street access may impact infection exposure. Likewise, the higher mean and median age of positive dogs compared with negative dogs may reflect a longer exposure time to potential environmental sources of infection for both males and females.

In the present study, the peridomiciliary presence of horses influenced the prevalence of dogs seroreactive for *Leptospira*. Interestingly, 214/320 (66.88%) horses used for carrying recycling material in the same urban area of the present study have shown seropositivity for leptospirosis (63), with 47/62 (75.80%) positive horses in a similar urban setting nearby, but there was no association with reactive dogs. These studies have suggested that seropositivity may be associated with horse permanence in low sanitary areas with the presence of rodents, similar to dog exposure and the likelihood of infection. However, as *Leptospira* strains have been isolated from mare urine (64), the possibility of infection in dogs from horse urine may not be ruled out and should be further investigated (65). Besides the relationship between environmental factors can be influence on this association, this factor was tested and not demonstrate significant results in the present study.

The results of this study were produced in a bivariate analysis. In addition, identification of which variables were significant was also relevant to explaining the dog leptospirosis prevalence in a multivariate context. Thus, four investigative econometric models have been estimated, including eventual neighborhood effects, meaning whether a dog has a higher likelihood of infection when the next-door neighbor dog is seropositive.

In addition, the internal conditions of the house do not have significant effects on animal infection. However, the parameters for the neighborhood were significant (Rho). Special attention should be given to the SAR and SEM models since eventual endogeneity problems are considered, and they use spatially lagged exogenous variables as instruments. These models may suggest that, if the neighbors' dogs have been infected, there would be an increased likelihood of infection of an animal in a specific household. Therefore, once again, the environment conditions, in addition to the residence, may be crucial to an increased probability of dog leptospirosis.

Only three variables were relevant in the explanation of the dependent variable, considering a 10% significance level. These results were interestingly similar to those observed in the logit model when not considering space. Dogs that have been vaccinated in the last 6 months are less likely to be infected, and if there was any individual with leptospirosis, the likelihood of an infected dog would be higher. The significance of the street variable suggests that the free-range dogs may be more likely to have a *Leptospira* infection than those who were bred indoors or were semidomiciled and finally, if a residence has an individual reactive to *Leptospira*, there is a greater probability that there will be an infected dog in the house.

The previous studies focused on the zoonotic infection with association of companion dog and owners has only been suggested in the presence of flooding areas as during an outbreak of hemorrhagic fever in late 1990's in Nicaragua (61) and after detection of *L. interrogans* in environmental water samples in Thailand (66), which has not occurred in the present study. Such findings may suggest a direct “flooding free” contact model involving a mammal triangle and cross-infection of owners and their dogs. The World Health Organization (WHO) authorities have already been alerted to the potential public health threat due to the increasing human:animal bond, especially due to zoonotic transmission suggesting a new global holistic and unified approach to One Health (67). Based on the comparative comprehensive study herein, strategies for control measures against leptospirosis should include pet vaccination, restriction of street access, and careful urine manipulation.

## Conclusion

Finally, the present study has shown a higher risk of owner leptospirosis associated with their own reactive dogs, particularly for serogroup Canicola, contributing to a better understanding of leptospirosis cross-species infection. In addition, simultaneous seropositivity in two owners living in the same household as their dogs strongly suggests an intradomicile-transmitted infection, with a direct or indirect role played by their owned dogs.

## Data Availability Statement

The raw data supporting the conclusions of this article will be made available by the authors, without undue reservation.

## Ethics Statement

The studies involving human participants were reviewed and approved by National Human Ethics Research Committee (protocol number 1,025,861/2014) at Londrina State University, Southern Brazil. The patients/participants provided their written informed consent to participate in this study. The animal study was reviewed and approved by Animal Use Ethics Committee (protocol number 181/2014), at Londrina State University, Southern Brazil. Written informed consent was obtained from the owners for the participation of their animals in this study.

## Author Contributions

AB contributed to conception, design of the study, and wrote sections of the manuscript. TM, AM, MR, IS, and TL contributed to organized the database. RF, CM, AB, and RR performed the statistical analysis and wrote sections of the manuscript. RM-B, JG, FC, EW, AK, and IN wrote sections of the manuscript. All authors contributed to manuscript revision, read, and approved the submitted version.

## Conflict of Interest

CM was employed by the company AAC&T Research Consulting LTDA. The remaining authors declare that the research was conducted in the absence of any commercial or financial relationships that could be construed as a potential conflict of interest.
