# Supplementary material for: Spatial and Simultaneous Seroprevalence of Anti-Leptospira Antibodies in Owners and Their Domiciled Dogs in a Major City of Southern Brazil
Source: Front Vet Sci. 2021 Jan 8;7:580400. doi: 10.3389/fvets.2020.580400 (PMC7820180; doi:10.3389/fvets.2020.580400)
Supplement: Supplementary file 1 [file Data_Sheet_1.docx]

**Supplementary information 1.a.** Matrix of the tetrameric correlations by the maximum likelihood estimator (iterative) obtained from the bivariate probit using the estimator as the initial value.

**Supplementary information 1.b.** Additional data extracted considering characteristic root criterion larger than one, as well as the highest percentage gains of the explained variable.

| **Factors** | **Characteristic roots** | **% of Variance** | |
| --- | --- | --- | --- |
|  |  | **Retained factors** | **Accumulated** |
| 1 | 2,33 | 38,5 | 38,5 |
| 2 | 1,31 | 21,7 | 60,2 |

**Supplementary information 1.c.** Factor loadings (pattern matrix) and unique variance

| **Variable** | **Factor 1** | **Factor 2** | **Uniqueness** |
| --- | --- | --- | --- |
| Wasteland | 0.16 | - | 0.97 |
| Outside bathroom | - | 0.29 | 0.90 |
| Presence of rats (3) | - | **0.72** | 0.27 |
| Yard cleaning | **0.66** | - | 0.36 |
| Rats seen in the backyard | - | **0.54** | 0.70 |
| Dirty backyard | **0.93** | - | 0.04 |
| Backyard with debris | **0.87** | - | 0.08 |

**Supplementary information 1.d.** Logit estimation without considering any spatial effects and controlling for the respective factors.

i.renda _Irenda_1-3 (naturally coded; _Irenda_1 omitted)

Iteration 0: log pseudolikelihood = -374.92451

Iteration 1: log pseudolikelihood = -364.91253

Iteration 2: log pseudolikelihood = -364.63957

Iteration 3: log pseudolikelihood = -364.63933

Iteration 4: log pseudolikelihood = -364.63933

Logistic regression Number of obs = 725

Wald chi2(11) = 20.69

Prob > chi2 = 0.0367

Log pseudolikelihood = -364.63933 Pseudo R2 = 0.0274

------------------------------------------------------------------------------

| Robust

LEPTO | Coef. Std. Err. z P>|z| [95% Conf. Interval]

-------------+----------------------------------------------------------------

Human leptospirosis | 1.242913 .4847005 2.56 0.010 .2929176 2.192909

Vaccination | -.6453717 .3547889 -1.82 0.069 -1.340745 .0500018

Street | .5469502 .2226836 2.46 0.014 .1104984 .983402

Income | -.0797113 .2317603 -0.34 0.731 -.5339531 .3745305

Income 3 | .1793953 .2717696 0.66 0.509 -.3532634 .7120539

Dog contact | -.3661105 .223448 -1.64 0.101 -.8040604 .0718395

Many dogs | .0478673 .0646837 0.74 0.459 -.0789104 .1746449

FAT_1 | -.5330865 .3299167 -1.62 0.106 -1.179711 .1135384

FAT_2 | -.495059 .3441676 -1.44 0.150 -1.169615 .1794971

pess_resid | .070609 .0600912 1.18 0.240 -.0471676 .1883857

TEMPONARES | -.0033627 .0070864 -0.47 0.635 -.0172518 .0105265

_cons | -1.224467 .3606107 -3.40 0.001 -1.931251 -.5176831

------------------------------------------------------------------------------

xi: logit LEPTO HL vacina1 rua i.renda contato_ca quant_caes FAT_1 FAT_2 pess_resid TEMPONARES, vce(robust)
